# Supplementary material for: Effect of a Multidimensional Physical Activity Intervention on Body Mass Index, Skinfolds and Fitness in South African Children: Results from a Cluster-Randomised Controlled Trial
Source: Int J Environ Res Public Health. 2019 Jan 15;16(2):232. doi: 10.3390/ijerph16020232 (PMC6352127; doi:10.3390/ijerph16020232)
Supplement: Supplementary file 1 [file ijerph-16-00232-s001.pdf]

Supplementary Material

Effect of a Multidimensional Physical Activity Intervention on Body Mass Index, Skinfolds and Fitness in South African Children: Results from a Cluster-Randomised Controlled Trial

Ivan Müller [1,2,3,*], Christian Schindler [1,2], Larissa Adams [4], Katharina Endes [3], Stefanie Gall [3], Markus Gerber [3], Nan S. N. Htun [1,2], Siphesihle Nqweniso [4], Nandi Joubert [4], Nicole Probst-Hensch [1,2], Rosa du Randt [4], Harald Seelig [3], Danielle Smith [4], Peter Steinmann [1,2], Jürg Utzinger [1,2], Peiling Yap [1,2,5], Cheryl Walter [4] and Uwe Pühse [3]

1. Swiss Tropical and Public Health Institute, P.O. Box, CH-4002 Basel, Switzerland; christian.schindler@swisstph.ch (C.S.); shwenwetun@gmail.com (N.S.N.H.); nicole.probst@swisstph.ch (N.P.-H.); peter.steinmann@swisstph.ch (P.S.); juerg.utzinger@swisstph.ch (J.U.); peiling.yap@gmail.com (P.Y.)
2. University of Basel, P.O. Box, CH-4003 Basel, Switzerland;
3. Department of Sport, Exercise and Health, University of Basel, Birsstrasse 320 B, CH-4052 Basel, Switzerland; ivan.mueller@unibas.ch (I.M.); katharina.endes@unibas.ch (K.E.); stefanie.gall@unibas.ch (S.G.); markus.gerber@unibas.ch (M.G.); harald.seelig@unibas.ch (H.S.); uwe.puehse@unibas.ch (U.P.)
4. Department of Human Movement Science, South Campus, P.O. Box 77000, Nelson Mandela University, Port Elizabeth 6031, South Africa; larissa.adams13@gmail.com (L.A.); felicitas.nqweniso@mandela.ac.za (S.N.); nandi.joubert@mandela.ac.za (N.J.); rosa.durandt@mandela.ac.za (R.d.R.); danielle.smith@mandela.ac.za (D.S.); cheryl.walter@mandela.ac.za (C.W.)
5. Institute of Infectious Disease and Epidemiology, Tan Tock Seng Hospital, 144 Moulmein Road, Singapore 308089, Singapore

* Correspondence: ivan.mueller@unibas.ch; Tel.: +41-61-207-4720; Fax: +41-61-207-4789

Supplementary Table S1. Descriptive results, stratified by physical activity intervention and control.

**Schools with physical activity intervention** (n=300; 3 schools, 11 classes)			**Schools without physical activity intervention** (n=446; 5 schools, 15 classes)	
**Baseline February 2015**	**Endline May 2016**	**Primary outcomes**	**Baseline February 2015**	**Endline May 2016**
300	278	BMI	446	405
300	272	Cardiorespiratory fitness	446	397
300	277	Average of six skinfolds	446	403
		**Secondary outcomes**		
300	277	Haemoglobin	446	401
300	300	Socioeconomic status	446	446
300	278	Self-reported physical activity	446	403
300	279	Stunting	446	402
300	264	Intestinal protozoa infection	446	374
300	264	STH infection	446	375

Supplementary Table S2. Intervention measures at eight primary schools in Port Elizabeth, South Africa.

Study arm	Schools with physical activity intervention	Schools without physical activity intervention
Physical activity	School 1	
Physical activity and health/hygiene education	School 2	
Physical activity, health/hygiene education and	School 3	
(No physical activity), health/hygiene education and nutrition education		School 4
(No physical activity)		Schools 5, 6, 7

Supplementary Table S3. Socioeconomic status among 746 schoolchildren aged 9–14 years from disadvantaged neighbourhoods in Port Elizabeth, South Africa in February 2015: Final results from the principal component analysis.

Variable description	Absolute frequency (N)	Relative frequency (P)	Coefficient in factor score
**Asset ownership**			
Washing machine for clothes	530	0.71	0.13
Fridge	680	0.91	0.19
Freezer for food	599	0.80	0.17
Radio	596	0.80	0.07
Land line phone	231	0.31	0.07
Television	723	0.97	0.10
Cell phone	721	0.97	0.10
Car	455	0.61	0.14
Computer	457	0.61	0.16
**Housing**			
Shack in informal settlement	86	0.12	−0.25
Backyard shack	36	0.05	−0.06
Privately built house	50	0.07	0.06
RDP house[a]	301	0.40	0.11
Council house	273	0.37	0.05
House of zinc	84	0.11	−0.22
House of bricks	630	0.84	0.26
House of wood	32	0.04	−0.12
Bathroom	422	0.57	0.24
Electricity inside the house	722	0.97	0.18
Toilet inside the house	496	0.67	0.27
Flush toilet	627	0.84	0.25
Pit toilet, bucket or communal toilet	119	0.16	−0.25
Tabs inside house	555	0.74	0.25
Tab in the yard	120	0.16	−0.13
Water tank or communal tab	71	0.10	−0.22
Cooking with electricity	697	0.93	0.18
Cooking with gas, paraffin stove or fire	49	0.07	−0.18

[a] In South Africa: Reconstruction and Development Programme house

Supplementary Table S4. Additional analysis according to a factorial design[a] achieved after exclusion of one of the schools. Estimated parallel effects of a) physical fitness intervention and b) combined health education and nutritional intervention on the mean changes in the respective outcome measures between baseline and endline.

Variables	Physical activity intervention[b]		Combination of health education and a nutritional intervention[c]		
	Estimated effect [d] (95% CI)	P-value	Estimated effect[d] (95% CI)	P-value	ICC[e]
**Cardiorespiratory fitness**					
Shuttle run (laps)	−0.69 (−5.20 to 3.81)	0.76	−4.83 (−9.48 to −0.18)	0.04	0.044
VO_2max[f] (ml×kg^{-1}×min^{-1})	−0.17 (−1.32 to 0.97)	0.76	−1.30 (−2.48 to −0.12)	0.03	0.038
**Obesity**					
BMIZ[g]	−0.16 (−0.24 to −0.09)	<0.001	−0.04 (−0.12 to 0.04)	0.28	0.00
Skinfolds[h] (mm)	−1.06 (−1.91 to −0.21)	0.02	0.67 (−0.19 to 1.54)	0.13	0.024
Mean in self-reported physical activity[i]	−1.05 (−2.36 to 0.26)	0.12	0.70 (−0.63 to 2.03)	0.3	0.055

[a] The two interventions distinguished in this analysis were a physical fitness intervention on the one hand (referred to as A) and the combination of a health education and a nutritional intervention (referred to as B) on the other hand. They were each represented by a binary factor in the analysis. After one school with physical fitness and health education intervention was excluded, there were 4 schools without any intervention and one school for each of the other combinations of the two factors (i.e., A, B and A+B)

[b] The physical fitness intervention consisted in physical education lessons taking place twice a week; weekly moving-to-music classes; in-class activity breaks; and school infrastructure adaptation to promote physical activity in two phases of 10 weeks each, as described in Figure 2 and it was applied in three of the eight schools between baseline and endline. One of these schools was excluded from the present analysis because the physical activity intervention was combined with the health education programme only and not with the combined health education and nutritional intervention

[c] The combined health education and nutritional intervention (referred to as factor B) consisted in a series of classroom-based lessons to help increase the awareness for intestinal parasite infections and education on treatment and prevention methods, such as proper hygiene, sanitation habits and consuming clean water/food and to help increase the awareness for healthy nutrition. Furthermore, an analysis of the school feeding programme was done and the cooks in the schools were trained in basic nutrition and hygiene during preparation of the school meals. The schoolchildren were given a ready to use supplementary food (RUSF) in the form of an enriched lipid-based paste. The combination of these two additional programmes was applied in two of the eight schools, once with additional application of the physical fitness intervention and once without

[d] Estimate of respective intervention effect on the change in the respective outcome measure from baseline to endline, with 95%-confidence interval. The underlying linear mixed models included binary factor variables for each of the two interventions A and B providing the respective effect estimates, along with baseline value of the respective outcome variable and baseline values of age, sex, height-for-age Z-score (HAZ), haemoglobin, socioeconomic (SES) index, protozoa- and soil-transmitted helminth (STH) infection status and random effects for classes and schools

[e] Proportion of unexplained variance attributable to clustering within schools and classes (ICC; intraclass correlation
coefficient)

[f] Using age-adjusted test protocol from Léger et al. [20]

[g] Sex-adjusted BMI-for-age Z-score (BMIZ)

[h] Average of six measurements (triceps and subscapular three times each)

[i] Score generated based on self-reported physical activity in the personal free time over the past 7 days outside school hours (range: from 1 to 14; 14 being the most active)

Supplementary Table S5. Cardiorespiratory fitness and obesity outcome measures from schoolchildren in Port Elizabeth, South Africa, at baseline (February 2015) and after a physical activity intervention (7-month midline; September 2015). Values are unadjusted means (standard deviations) unless specified otherwise and estimated effects of the physical activity intervention on the mean changes in the respective outcome measures between baseline and midline.

Variables	Schools with physical activity intervention		Schools without physical activity intervention		Intervention effect[a]		
	Baseline (n=300)	Midline (n=294)	Baseline (n=446)	Midline (n=417)	Estimate[b] (95% CI)	P-value	ICC[c]
**Cardiorespiratory fitness**							
Shuttle run (laps)	35.6 (17.0)	32.3 (18.4)	36.8 (17.4)	33.9 (18.2)	−1.53 (−4.59 to 1.53)	0.33	0.02
VO_2max[d] ($ml \times kg^{-1} \times min^{-1}$)	45.8 (4.1)	43.9 (4.7)	46.3 (4.3)	44.6 (4.7)	−0.54 (−1.31 to 0.22)	0.16	0.01
**Obesity**							
BMIZ[e]	−0.1 (1.2)	0.1 (1.1)	0.0 (1.2)	0.2 (1.2)	−0.01 (−0.08 to −0.05)	0.73	0.01
Skinfolds[f] (mm)	9.0 (4.5)	8.9 (4.3)	9.0 (4.4)	10.5 (6.5)	−1.68 (−2.61 to −0.75)	<0.001	0.02
Mean of self-reported physical activity[g]	9.1 (3.7)	10.2 (2.9)	7.8 (3.8)	9.0 (3.3)	0.29 (−0.83 to 1.40)	0.61	0.10

[a] Schoolchildren from the intervention group accomplished a multidimensional physical activity intervention programme between baseline and midline, as described in Figure 2; Estimate of the physical activity intervention effect on the change in the respective outcome measure from baseline to midline, with 95%-confidence interval. The underlying linear mixed models included binary factor variables for all three intervention programmes (i.e., the physical activity intervention, the health education and the nutrition education programme) providing the physical activity intervention effect estimates, included baseline value of the respective outcome variable and baseline values of age, sex, height-for-age Z-score (HAZ), haemoglobin, socioeconomic (SES) index, protozoa- and soil-transmitted helminth (STH) infection status and random effects for classes and schools

[b] Estimate of respective intervention effect on the change in the respective outcome measure from baseline to midline, with 95%-confidence interval, P-value and ICC. The underlying linear mixed models included binary factor variables for each of the two interventions A (physical fitness intervention) and B (combination of a health education and a nutritional intervention) providing the respective effect estimates, along with baseline values of age, sex, HAZ, haemoglobin, SES index, protozoa- and STH infection status and random effects for classes and schools

[c] Proportion of unexplained variance attributable to clustering within schools and classes (intraclass correlation coefficient; ICC)

[d] Using age-adjusted test protocol from Léger et al. [20]

[e] Sex-adjusted BMI-for-age Z-score (BMIZ)

[f] Average of six measurements (triceps and subscapular three times each)

[g] Score generated based on self-reported physical activity in the personal free time over the past 7 days and intense exercises outside structured school hours (range: from 1 to 14; 14 being the most active)

Supplementary Table S6. Cardiorespiratory fitness and obesity outcome measures from schoolchildren in Port Elizabeth, South Africa, at midline (September 2015) and after a physical activity intervention (16-month endline; May 2016). Values are unadjusted means (standard deviations) unless specified otherwise and estimated effects of the physical activity intervention on the changes in the respective outcome measures between midline and endline.

**Variables**	**Schools with physical activity intervention**		**Schools without physical activity intervention**		**Intervention effect**[a]		
	**Midline (n=294)**	**Endline (n=264)**	**Midline (n=417)**	**Endline (n=255)**	**Estimate**[b] **(95% CI)**	**P-value**	**ICC**[c]
**Cardiorespiratory fitness**							
Shuttle run (laps)	32.3 (18.4)	34.5 (17.9)	33.9 (18.2)	35.3 (18.7)	2.88 (−0.42 to 6.17)	0.09	0.004
VO_2max[d] ($ml \times kg^{-1} \times min^{-1}$)	43.9 (4.7)	43.5 (4.7)	44.6 (4.7)	44.0 (4.8)	0.92 (0.10 to 1.73)	0.03	<0.001
**Obesity**							
BMIZ[e]	0.1 (1.1)	−0.1 (1.3)	0.2 (1.2)	0.2 (1.3)	−0.16 (−0.25 to −0.07)	0.001	0.02
Skinfolds[f] (mm)	8.9 (4.3)	9.6 (4.6)	10.5 (6.5)	10.1 (5.9)	0.51 (−0.25 to 1.28)	0.19	0.03
Mean of self-reported physical activity[g]	10.2 (2.9)	9.0 (3.1)	9.0 (3.3)	9.9 (3.4)	−1.11 (−2.39 to 0.18)	0.09	0.07

[a] Schoolchildren from the intervention group accomplished a multidimensional physical activity intervention programme between midline and endline, as described in Figure 2; The underlying linear mixed models included binary factor variables for all three intervention programmes (i.e., the physical activity intervention, the health education and the nutrition education programme) providing the physical activity intervention effect estimates, included midline value of the respective outcome variable and midline values of age, sex, height-for-age Z-score (HAZ), haemoglobin, socioeconomic (SES) index, protozoa- and soil-transmitted helminth (STH) infection status and random effects for classes and schools

[b] Estimate of respective intervention effect on the change in the respective outcome measure from midline to endline, with 95%-confidence interval, P-value and ICC. The underlying linear mixed models included binary factor variables for each of the two interventions A (physical fitness intervention) and B (combination of a health education and a nutritional intervention) providing the respective effect estimates, along with midline values of age, sex, HAZ, haemoglobin, SES index, protozoa- and STH infection status and random effects for classes and schools

[c] Proportion of unexplained variance attributable to clustering within schools and classes (intraclass correlation coefficient; ICC)

[d] Using age-adjusted test protocol from Léger et al. [20].

[e] Sex-adjusted BMI-for-age Z-score (BMIZ)

[f] Average of six measurements (triceps and subscapular three times each)

[g] Score generated based on self-reported physical activity in the personal free time over the past 7 days and intense exercises outside structured school hours (range: from 1 to 14; 14 being the most active)
